# Light adaptation does not prevent early retinal abnormalities in diabetic rats

**DOI:** 10.1038/srep21075

**Published:** 2016-02-08

**Authors:** Joanna Kur, Michael A. Burian, Eric A. Newman

**Affiliations:** 1 Department of Neuroscience, University of Minnesota, Minneapolis, MN 55455, USA

## Abstract

The aetiology of diabetic retinopathy (DR), the leading cause of blindness in the developed world, remains controversial. One hypothesis holds that retinal hypoxia, exacerbated by the high O_2_ consumption of rod photoreceptors in the dark, is a primary cause of DR. Based on this prediction we investigated whether early retinal abnormalities in streptozotocin-induced diabetic rats are alleviated by preventing the rods from dark adapting. Diabetic rats and their non-diabetic littermates were housed in a 12:12 hour light-dim light photocycle (30 lux during the day and 3 lux at night). Progression of early retinal abnormalities in diabetic rats was assessed by monitoring the ERG b-wave and oscillatory potentials, Müller cell reactive gliosis, and neuronal cell death, as assayed by TUNEL staining and retinal thickness at 6 and 12 weeks after diabetes induction. Maintaining diabetic animals in a dim-adapting light did not slow the progression of these neuronal and glial changes when compared to diabetic rats maintained in a standard 12:12 hour light-dark photocycle (30 lux during the day and 0 lux at night). Our results indicate that neuronal and glial abnormalities in early stages of diabetes are not exacerbated by rod photoreceptor O_2_ consumption in the dark.

Diabetic retinopathy (DR) is a serious complication of both type 1 and type 2 diabetes and a leading cause of visual impairment and blindness^1^. With the current epidemic of type 2 diabetes, DR will become an even more serious health problem in the coming decades.

The initial steps leading to DR have not been fully elucidated. One hypothesis of the aetiology of DR holds that compromised blood flow in early stages of the disease results in retinal hypoxia. Although the evidence for hypoxia in DR is controversial, some studies support the hypothesis. In psychophysical experiments on diabetic patients, reduced contrast sensitivity and reduced colour vision perception is restored when patients breath 100% O_2_^2^,^3^. In animal models of diabetes, pimonidazole labelling^4^, the expression of hypoxia-inducible factor-1^5^, and intraretinal PO_2_ profiles in cats diabetic for 6 years^6^ all indicate retinal hypoxia.

GB Arden has suggested that the high metabolic demand of photoreceptors in the dark will exacerbate retinal hypoxia and may be a primary cause of DR^7^. He has proposed that preventing the rod photoreceptors from dark adapting, which will significantly reduce their metabolic demand, may lessen hypoxia and slow progression of DR. In support of this hypothesis, Arden has found in preliminary trials on diabetic patients that light exposure during the night to prevent the rods from dark adapting results in the improvement of visual function and regression of macular oedema in diabetic patients^8^,^9^.

Diabetic retinopathy has traditionally been viewed as a disease of the retinal vasculature. However, there is strong evidence that neuronal and glial dysfunction precede overt vascular lesions and play a role in the development of DR^10^. In both patients and in animal models of diabetes there is a loss of retinal neurons early in disease progression^11^. Neuronal dysfunction is reflected in alterations in the electroretinogram (ERG)^12^,^13^. Moreover, in response to retinal hypoxia, Müller cells (the principal glial cells of the retina) are activated and upregulate proangiogenic and vascular permeability factors such as vascular endothelial growth factor (VEGF)^10^,^14^, which may contribute to the development of clinical symptoms of retinopathy.

We have now assessed whether preventing dark-adaptation reduces early neuronal and glial changes in the diabetic retina in a rat model of type 1 diabetes. We find that preventing dark-adaptation by maintaining diabetic rats in dim light during the night does not slow the progression of early retinal abnormalities, as assessed by changes in the ERG, Müller cell reactive gliosis, and neuronal cell death, assayed by TUNEL staining and retinal thickness.

## Results

We tested the hypothesis that preventing dark adaptation slows the progression of early retinal abnormalities in diabetic rats using the streptozotocin (STZ) animal model of type 1 diabetes. Sprague-Dawley rats were treated with a single injection of STZ or citrate buffer and serum glucose was assessed weekly. STZ-treated rats became hyperglycaemic within 3 days after injection, and glucose concentrations remained elevated throughout the study (Table 1). Rats were housed in a 12:12 light-dark or light-dim light photocycle. Animals housed under standard light-dark conditions were maintained at 30 lux–0 lux while animals housed under dim-adapting conditions were maintained at 30 lux–3 lux. Rats were assessed for progression of retinal abnormalities at 6 and 12 weeks following diabetes induction.

### Effects of dim background light on the electroretinogram

Rats housed under dim-adapting conditions were maintained in a room with an ambient light intensity within the cages of 3.0 ± 0.9 lux during subjective night to prevent rod photoreceptor dark adaptation. We assessed whether 3 lux illumination was sufficient to light-adapt the rods by measuring changes in the ERG under different background illumination levels.

**Figure 1:**
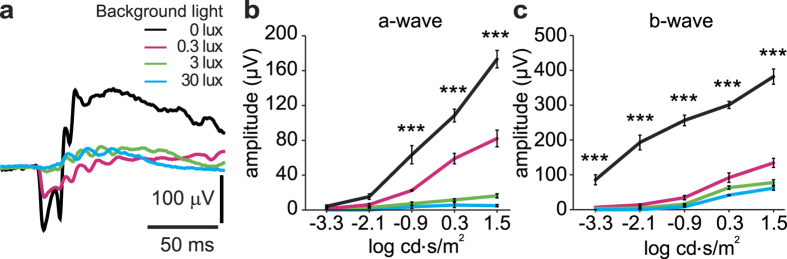
Effect of background illumination on the ERG in control (non-diabetic) rats housed under standard lighting conditions. (**a**) ERG responses to flash stimuli of 1.50 log cd.s/m^2^ recorded under dark-adapted (0 lux background) and dim- and light-adapted conditions (background light intensities of 0.3, 3 and 30 lux). (**b**,**c**) Intensity-response relations for the a-wave (**b**) and the b-wave (**c**) under different background illuminance conditions. N = 5 rats. Background light levels as low as 0.3 lux light adapt the retina. ***P < 0.001 for multiple comparisons between ‘0 lux’ and background light intensities of 0.3, 3 and 30 lux. Error bars here and in other figures denote ± s.e.m.

Flash ERGs were recorded in control (non-diabetic) rats in the dark (0 lux) and at three background light intensities (0.3, 3 and 30 lux). Both the a-wave and b-wave were significantly reduced with background light levels as low as 0.3 lux (Fig. 1a–c), demonstrating that this dim-adapting illumination light adapts the retina.

### Evaluation of retinal abnormalities

We evaluated the progression of diabetes-induced retinal abnormalities in rats housed under standard and dim-adapted lighting conditions. Changes in the ERG, Müller cell reactive gliosis, neuronal apoptosis, and reduced retinal thickness, a measure of retinal cell loss, were measured.

#### The electroretinogram

**Figure 2:**
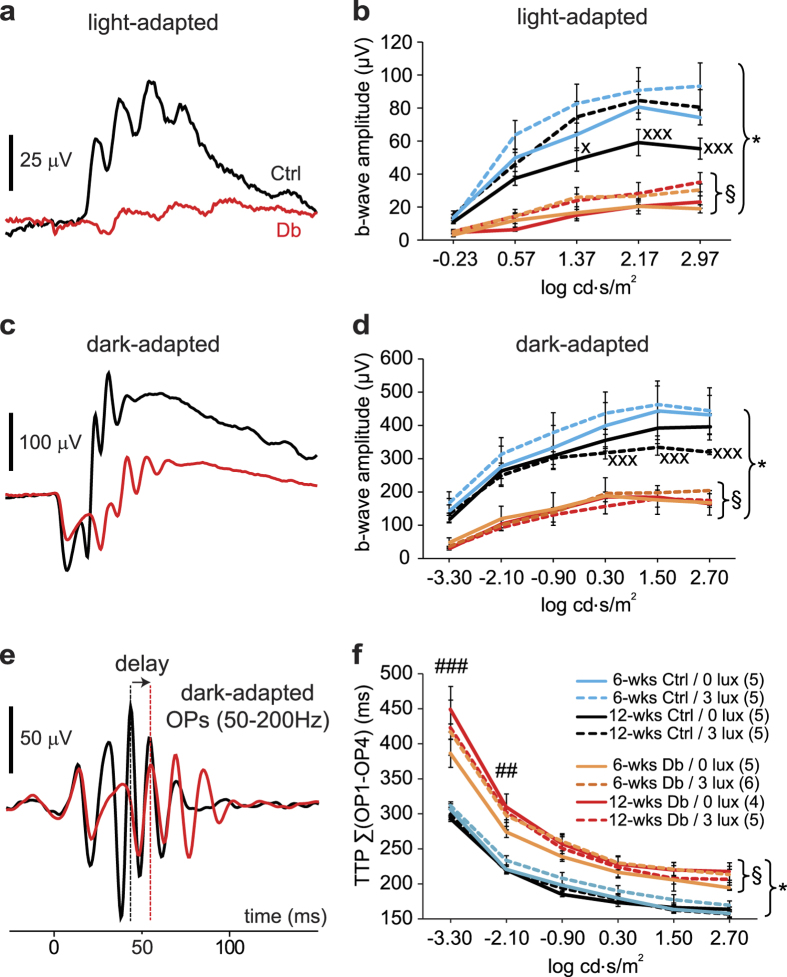
Electroretinograms from control and diabetic rats. Left panels show representative ERG waveforms from rats diabetic for 12 weeks (red traces) and aged-matched controls (black traces) housed under standard lighting conditions. Right panels show summary intensity-response relations. (**a**,**b**) Light-adapted retinas. Responses in (**a)** are to a flash at 2.17 log cd.s/m^2^. b-Wave amplitudes are shown in (**b)**. (**c**,**d**) Dark-adapted retinas. Responses in (**c)** are to a flash at 1.50 log cd.s/m^2^. b-Wave amplitudes are shown in (**d)**. (**e**,**f**) Oscillatory potentials. Responses in (**e)** are to a flash at 1.50 log cd.s/m^2^. The sum of the oscillatory potentials time-to-peak (TTP) for OP_1_ through OP_4_ are shown in (**f**) In the right panels, rats diabetic for 6 and 12 weeks (Db) and aged-matched controls (Ctrl), housed under standard light conditions (0 lux, continuous traces) or dim-adapted at night conditions (3 lux, dashed traces) are shown. Diabetic rats had reduced b-wave amplitudes and increased latency of OPs at both 6 and 12 weeks of diabetes, compared to controls. Housing conditions (standard vs. dim-adapting) did not affect b-wave amplitude or oscillatory potential delay. Numbers in parentheses indicate number of rats. §, not significant for comparison among diabetic groups; *P < 0.05, for comparison between each diabetic group and aged-matched control. ^##^P < 0.01 and ^###^P < 0.001 for comparison between standard diabetic rats at 6- and 12-weeks. ^x^P < 0.05 and ^xxx^P < 0.001 for comparison between control groups at 6- and 12-weeks (standard in (**b)**, and dim-adapted in (**d)**).

The ERG b-wave, reflecting bipolar cell function, and the oscillatory potentials (OPs), primarily reflecting the activity of amacrine cells^15^, were recorded. These ERG potentials show characteristic changes in early stages of retinal pathology in diabetic patients and in diabetic animals^12^,^13^. Our results were consistent with previous studies. There was a significant reduction in the amplitude of the light- and dark-adapted b-wave as early as 6 weeks following diabetes induction, compared with aged-matched control rats (Fig. 2a–d). In addition, diabetic rats showed significantly delayed OP responses starting at 6 weeks of diabetes (Fig. 2e,f).

However, there were no differences in ERG responses between diabetic rats housed under standard and dim-adapted lighting conditions. Changes in b-wave amplitudes and in OP delays were similar in the two groups of animals.

#### Müller cell gliosis

Müller cell reactive gliosis is a hallmark of retinal disease and is observed in diabetic patients and in animal models of diabetes^5^,^16^. We assessed the expression of the intermediate filament, glial fibrillary acidic protein (GFAP), in transverse sections of the retina to evaluate the progression and severity of Müller cell reactivity.

**Figure 3:**
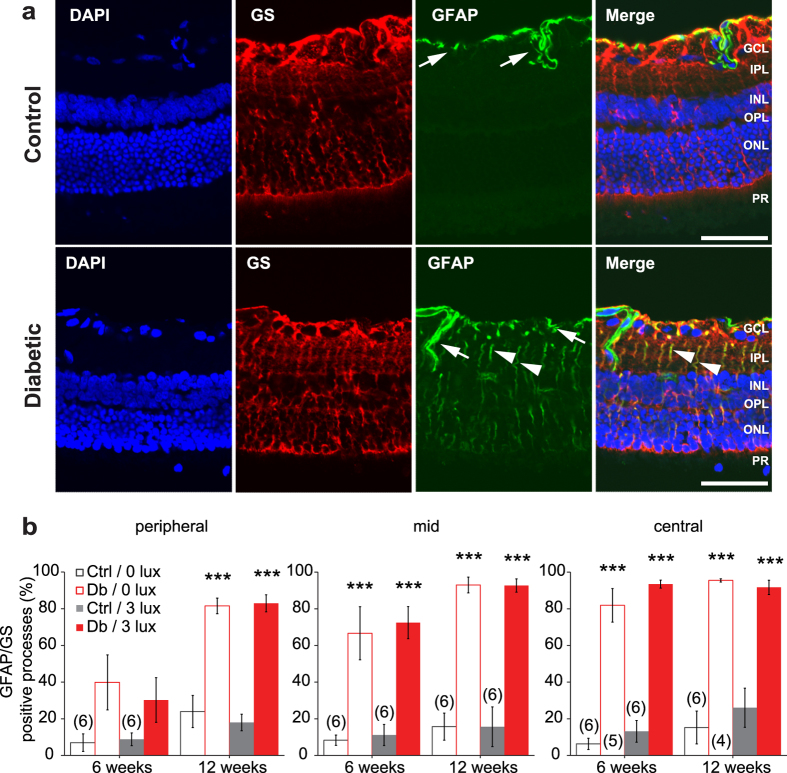
Müller cell reactive gliosis. (**a**) Immunohistochemical labelling for the Müller cell specific enzyme glutamine synthetase (GS, red) and for GFAP (green) from rats diabetic for 12 weeks (bottom panels) and aged-matched controls (top panels) housed under standard lighting conditions. The nuclear stain DAPI is shown in blue. In control retinas Müller cells showed little labelling for GFAP, with expression restricted to astrocytes (arrows) at the retinal surface and along penetrating vessels. In diabetic rats, Müller cells displayed increased gliosis (GS-positive processes that were also GFAP-positive; arrowheads). For this and Fig. 4, GCL, ganglion cell layer; IPL, inner plexiform layer; INL, inner nuclear layer; OPL, other plexiform layer, ONL, outer nuclear layer; PL, photoreceptor layer. Scale bar: 50 μm. (**b**) Summary data for Müller cell gliosis (percentage of GS-positive processes in the IPL that were also GFAP positive), at 6 and 12 weeks in peripheral, mid and central retina in control (Ctrl) and diabetic rats (Db) housed under standard (0 lux) or dim-adapted at night (3 lux) conditions. Müller cells in the mid and central retina display increased gliosis at 6 and 12 weeks of diabetes, and in the peripheral retina at 12 weeks. Housing conditions (standard or dim-adapting) did not affect the degree of gliosis at either 6 or 12 weeks. Numbers in parentheses indicate number of rats. ***P < 0.001 compared to aged-matched control.

Müller cell gliosis, evidenced by an upregulation of GFAP, was apparent at 6 weeks of diabetes in the central and mid retina, and at 12 weeks in the peripheral retina (Fig. 3). Control (non-diabetic) animals showed little gliosis. However, the percentage of Müller cells that expressed GFAP was similar for diabetic rats housed under standard and dim light conditions.

#### Cell death and retinal thickness

**Figure 4:**
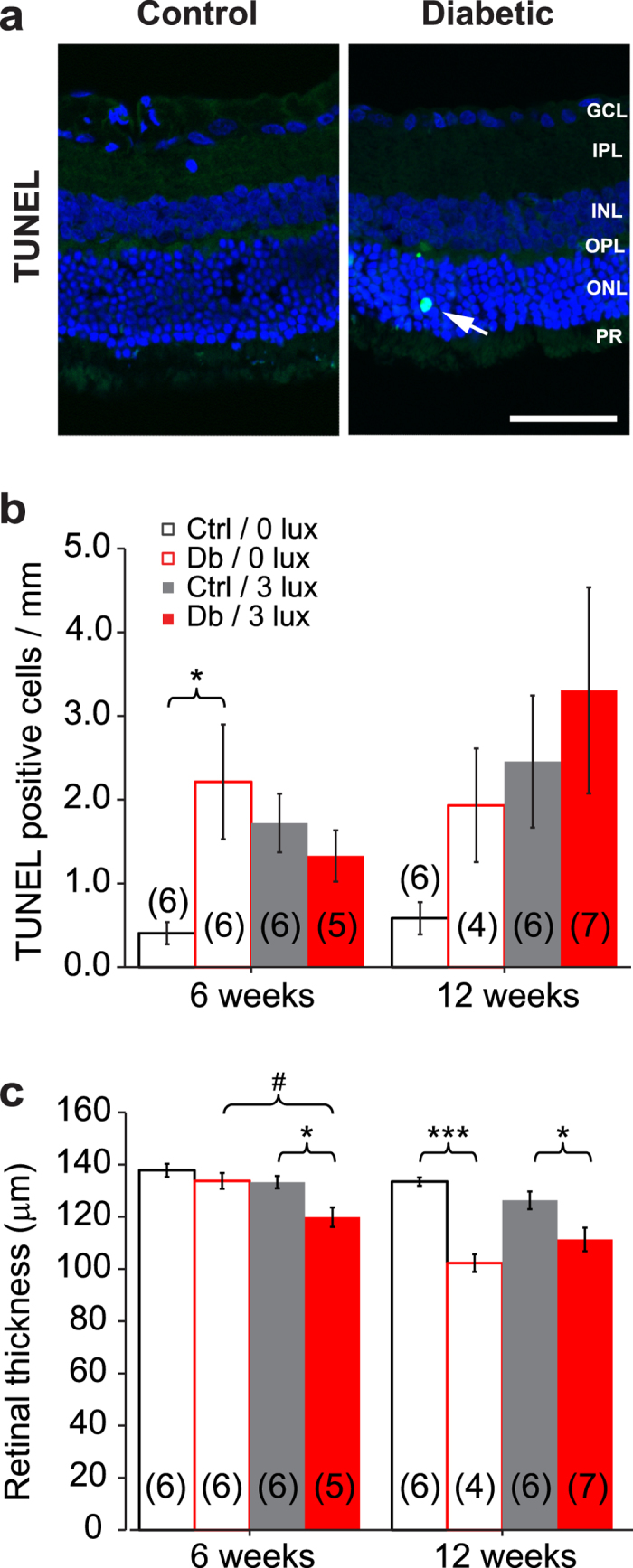
Cell death in control and diabetic retinas. (**a**) TUNEL staining in retinal cross-sections from rats diabetic for 6 weeks and aged-matched controls, housed under standard lighting conditions. DAPI-labelled nuclei (blue) and a TUNEL-positive cell (blue/green; arrow) are shown. Scale bar: 50 μm. (**b**) Mean number of TUNEL-positive cells from 12-micron-thick retinal sections through the optic disc, normalized to the length of the retinal slice. (**c**) Total retinal thickness in control (Ctrl) and diabetic rats (Db), housed under standard (0 lux) or dim-adapted at night (3 lux) conditions. Numbers in parentheses indicate number of rats. *P < 0.05, ***P < 0.001 vs age-matched control; ^#^P < 0.05 comparison between dim-adapted diabetic rats at 6- and 12-weeks.

Neuronal cell death, indicated by TUNEL staining, and the resulting decrease in retinal thickness, is observed in both diabetic patients and in STZ-treated diabetic rats^11^. We observed a significant increase in TUNEL-positive cells in retinas of 6 week diabetic rats (Fig. 4a,b). Approximately eighty-six percent of these TUNEL-positive cells were observed in the outer nuclear layer. Morphological assessment of retinal sections revealed a decrease in the thickness of the retina in diabetic rats at 6 weeks (3 lux only) and at 12 weeks (both diabetic groups) (Fig. 4c).

Diabetic rats housed under standard and dim light conditions showed similar increases in TUNEL-positive cells and similar decreases in retinal thickness. The one exception was retinal thickness at 6 weeks, where rats housed under dim light conditions showed a decrease in retinal thickness while rats housed under standard light conditions did not. This result might be due to light damage caused by the 3 lux dim-adapting light. At 12 weeks, reduced retinal thickness was apparent in both diabetic groups.

## Discussion

We have evaluated whether preventing dark adaptation with a dim adapting light slows the development of early neuronal and glial abnormalities in diabetic rats. Our results indicate that the development of these early diabetes-induced retinal abnormalities is not slowed, at least in our animal model of diabetes.

We found that diabetic rats housed under dim-adapting lighting conditions at night to prevent rod dark adaptation showed a similar degree of retinal abnormalities as rats housed under standard (0 lux) lighting conditions at night. This was true at both 6 and 12 weeks following diabetes induction and for multiple measures of retinal function and morphology, including ERG alterations, Müller cell reactive gliosis, and cell death, as assayed by TUNEL staining and retinal thickness. It should be noted, however, that neuronal cell loss in diabetes occurs predominately in the inner retina^17^,^18^. Thus, the cell loss we observed, largely in the photoreceptors, might not be relevant to the development of DR.

There are several possible reasons why light adaptation failed to slow the progression of retinal abnormalities. The 3 lux adapting light that was used to prevent rod dark adaptation at night may not have been bright enough to sufficiently reduce photoreceptor metabolism. This is unlikely, however, as a 0.3 lux background light was sufficient to cause significant reductions in retinal sensitivity, as measured by ERG a- and b-wave amplitudes. In addition, the 3 lux adapting light we used was similar in intensity to the adapting light used by Arden in his clinical trials on diabetic patients^8^,^9^. In his DR study from 2010^8^, a glow patch next to the eye was used to light-adapt the retina. The glow patch had a luminance of ~10 cd/m^2^. Accounting for the 0.3% transmittance of the human eyelid for green light^19^, this is equivalent to an illuminance of 0.3 lux at the surface of the globe.

Negative results may also have been obtained because the maximal diabetic duration of 12 weeks used in our study may have been too short to detect differences between experimental groups. In addition, the adapting light we used might not have reached the rat retina due to eye closure or burrowing. This is unlikely, however, because rats are active during the subjective night, even when exposed to bright background light^20^,^21^.

The focus of our study was to evaluate neuronal and glial abnormalities in the retina in early stages of diabetes. Vascular changes, which occur in diabetic rats only after many months, were not followed. It is possible that the light adaptation protocol we used to reduce retinal hypoxia might have been beneficial in limiting the vascular complications of DR, as vascular abnormalities might be caused by different mechanisms than neuronal and glial abnormalities. Species differences in retinal PO_2_ also complicate the interpretation of our results. Minimum retinal PO_2_ in the dark is lower in primates than it is in rodents^22^,^23^. Thus, light adaptation might have a larger effect in preventing hypoxia in humans than it does in rats.

The dim-adapting light used in our experiments would be expected to reduce oxidative stress and the production of free radicals by photoreceptors, which may contribute to the development of DR^24^. However, our study did not evaluate the effect of light therapy on oxidative stress in the retina.

Our results indicate that preventing dark adaptation and thus reducing retinal O_2_ consumption does not slow the development of early neuronal and glial abnormalities in diabetic rat retinas. This may occur because the diabetic rat retina may not, in fact, be hypoxic^25^,^26^. Notably, measurements of intraretinal PO_2_ in diabetic rats at 12 weeks following diabetes induction indicate an increase, not a decrease in PO_2_^27^.

Although our results indicate that early diabetes-induced neuronal and glial abnormalities are not caused by retinal hypoxia exacerbated by rod O_2_ consumption, the results do not rule out the dark adaptation-hypoxia hypothesis of DR. The validity of this hypothesis will only be resolved by additional experimentation. Ultimately, large scale clinical trials will determine whether dim-adapting light therapy is beneficial in slowing the progression of DR. One such clinical study is currently being conducted^28^.

## Methods

### Induction of Diabetes

All experimental procedures were approved by and adhered to the guidelines of the Institutional Animal Care and Use Committee of the University of Minnesota (Protocol ID: 1304-30570A). Two-month-old Sprague-Dawley male rats received a single intraperitoneal (i.p.) injection of streptozotocin (STZ; 70 mg/kg dissolved in 0.01 M citrate buffer, pH 4.5) or vehicle alone. Successful induction of diabetes, defined as a blood glucose level ≥250 mg/dl, was confirmed 3 days later. Non-fasting blood glucose and weight were monitored weekly with a OneTouch Ultra Glucometer (Life Scan, USA) and, if required, diabetic rats received insulin (Lantus insulin glargine, SANOFI, subcutaneously, thrice a week) at a sub-therapeutic dose of either 1.5 U (glucose range of 250–400 mg/dl) or 2.5 U (above 400 mg/dl) to prevent excessive weight loss and a catabolic response.

### Housing conditions

Rats were randomly assigned to two experimental groups. One group of animals was housed under a 12:12 light-dark photocycle with illumination of 30 lux during the day and 0 lux at night (standard lighting). A second group was housed under a 12:12 light-dim light photocycle of 30 lux during the day and 3 lux at night (dim-adapting lighting). Dim illumination conditions were achieved with two 4 Watt compact fluorescent light bulbs (colour temperature 2700 K). These housing conditions were introduced three days before induction of diabetes and maintained for the duration of the experiment. Light levels were measured with a digital illuminance meter (Easy View Light Meter, ExTech Instruments) in the centre of the polycarbonate cages used to house the animals. The 3 lux night illumination was used to prevent dark adaptation. This intensity corresponds to a luminance level in the mid-mesopic range^29^ and was chosen as likely to be below the intensity causing phototoxic damage in healthy albino rats^30^. All rats were allowed free access to food and water.

In addition, five untreated male Sprague-Dawley rats (2 months old, weighting ~300 g) were used to assess the effects of background illumination on the ERG. Rats from this group were housed under standard lighting conditions.

### The electroretinogram

Rats were maintained in complete darkness overnight prior to an ERG recording session. Preparations for recordings were conducted under dim red illumination and were followed by a 20 minute period of complete darkness to ensure a dark-adapted state. The full-field flash ERG was recorded using custom DTL fibre electrodes from isoflurane-anesthetized rats (1.5%–2% in 24% O_2_/76% N_2_), with an Espion testing system and ColorDome LED/Xenon-full field stimulator (Diagnosys LLC, Lowell, MA). Pupils were dilated with 1% atropine (Akorn, Inc. IL) and the corneal surface anesthetized with 0.5% proparacaine HCl (Bausch&Lomb,Inc. FL). Eyes were lubricated with 2.5% Hypermellose (Gonivisc, HUB Pharmaceuticals). After completing a scotopic intensity series, photopic flash responses were recorded in presence of an adapting white light (30 cd/m^2^, photopic units). Flash ERG records were band pass filtered at 0.3 and 500 Hz. Oscillatory potentials were band pass filtered at 50–200 Hz. Each record represents an average of 2–6 responses. Rats were killed immediately following ERG measurement and eyes were harvested for histological evaluation.

### Immunohistochemistry

Whole retinas were fixed in 4% paraformaldehyde in PBS for 45 min and shock-frozen in a 50–50% mixture of OCT compound (Sakura Tissue-Tek) and Aquamount (Lerner Laboratories). Twelve-μm-thick cryostat sections were cut and mounted using Vectashield containing DAPI (Vector Laboratories). Sections were imaged with confocal microscopy (Olympus FluoView 1000), using either a 20X oil (UPlan SApo, 0.85 NA) or a 40X oil (UPlan Fl N, 1.30 NA) objective. Offset, laser power and gain parameters were held constant for control and diabetic samples.

To assess Müller cell gliosis, transverse retinal sections were double-labelled with antibodies to glial fibrillary acidic protein (GFAP) and glutamine synthetase (GS), a Müller cell-specific marker, using procedures modified from^5^. Nonspecific binding was blocked with 10% normal donkey serum for 1 hour at room temperature. Tissue samples were incubated with primary antibodies overnight at 4 deg C (GFAP, 1:500, goat anti-GFAP, Santa Cruz and GS, 1:200 mouse anti-GS, BD Biosciences) and secondary antibodies (1:500, donkey anti-mouse Alexa Fluor 647 and donkey anti-goat Alexa Fluor 488; Molecular Probes) for 2 hours at room temperature. The percentage of GS-positive Müller cell processes in the inner plexiform layer that were co-labelled for GFAP were counted. For each section, measurements were made in three retinal regions ~800 microns apart, one near the optic disc (central), a second in the mid retina and a third in the periphery. All immunostainings were repeated twice. Staining that omitted the primary antibody served as a negative control.

Total thickness in the central region of the retina was evaluated in DAPI-stained cryostat sections that crossed the optic disc. Although histological artifacts such as dehydration and shrinkage can contribute to changes in the structure of histological sections, these processes will affect the measurement of retinal thickness equally in all experimental groups.

### TUNEL labelling

Apoptotic cells were imaged with TUNEL labelling using the *In Situ* Cell Death Detection Kit, Fluorescein (Roche, Basel, Switzerland) according to the protocol provided by the supplier. Briefly, fixed sections were permeabilized in PBS containing Triton X-100 (Sigma) and then incubated in the reaction mixture for 60 min at 37 deg C. TUNEL-positive cells were counted manually in each of two sections running through the optic disc and analysed as the number of positive cells per length of the retinal section.

### Statistical Analysis

Experimental values are reported as mean ± s.e.m. Statistical analysis was performed using GraphPad Prism software. Standard and repeated measures ANOVA was used to compare experimental groups across time points and housing conditions. Post hoc multiple comparisons were performed when appropriate using Tukey’s method. Two-sided t-test was used to determine pairwise differences. All analyses were performed with significance at P < 0.05.

## Additional Information

**How to cite this article**: Kur, J. *et al.* Light adaptation does not prevent early retinal abnormalities in diabetic rats. *Sci. Rep.* **6**, 21075; doi: 10.1038/srep21075 (2016).

## Figures and Tables

**Table 1:** Blood glucose and body weight of experimental rats.

		Standard lighting	Dim-adapting	Control	Diabetic
		Control	Diabetic		
Glucose (mg/dl)	0 weeks	117.8 ± 5.1 (13)	119.2 ± 7.6 (9)	110.5 ± 2.2 (12)	113.8 ± 4.2 (13)
	3 days	103.4 ± 3.3^#^ (13)	551.9 ± 17.8^***^(9)	105.2 ± 2.5 (12)	551.9 ± 15.6^***^ (13)
	6 weeks	100.4 ± 2.6^##^ (13)	511.6 ± 23.5^***^ (9)	99.7 ± 6.2 (6)	492.8 ± 32.9^***^ (13)
	12 weeks	100.7 ± 3.2 (7)	452 ± 32.3^***^ (4)	92.8 ± 4.3 (6)	438.9 ± 39.5^***^ (7)
Weight (g)	0 weeks	288.0 ± 5.2 (13)	282.4 ± 2.4 (9)	295.0 ± 6.2 (12)	291.5 ± 5.3 (13)
	6 weeks	399.3 ± 5.0^###^ (13)	246.8 ± 9.0^***,#^ (9)	402.1 ± 5.5^###^ (12)	274.9 ± 13.7^***^ (13)
	12 weeks	459.7 ± 6.5^###^ (7)	255.8 ± 18.4^***^ (4)	460.0 ± 14.4^###^ (6)	303.4 ± 28.0^***^ (7)

STZ-treated rats were hyperglycaemic (≥250 mg/mL) from day 3 onward. While healthy rats (both control groups) gained weight over the 12-weeks period, diabetic rats housed under standard light conditions lost weight and dim-adapted diabetic rats did not change. ***P < 0.001 compared to age-matched controls; ^#^P < 0.05, ^##^P < 0.01 and ^###^P < 0.001 compared to ‘0 weeks’ within same treatment group. Data are expressed as the mean ± s.e.m. with sample size in parentheses.
